# Tunable random lasing behavior in plasmonic nanostructures

**DOI:** 10.1186/s40580-016-0095-5

**Published:** 2017-01-09

**Authors:** Ashish Yadav, Liubiao Zhong, Jun Sun, Lin Jiang, Gary J. Cheng, Lifeng Chi

**Affiliations:** 1 Institute of Functional Nano and Soft Materials (FUNSOM), Soochow University, Suzhou, 215123 Jiangsu People’s Republic of China; 2 School of Industrial Engineering, Purdue University, 315 N. Grant St, West Lafayette, IN 47907 USA; 3 Birck Nanotechnology Center, Purdue University, 1205 W State St, West Lafayette, IN 47907 USA

**Keywords:** Plasmonics, Nanomaterials, Scattering, Surface plasmons and random lasing

## Abstract

Random lasing is desired in plasmonics nanostructures through surface plasmon amplification. In this study, tunable random lasing behavior was observed in dye molecules attached with Au nanorods (NRs), Au nanoparticles (NPs) and Au@Ag nanorods (NRs) respectively. Our experimental investigations showed that all nanostructures i.e., Au@AgNRs, AuNRs & AuNPs have intensive tunable spectral effects. The random lasing has been observed at excitation wavelength 532 nm and varying pump powers. The best random lasing properties were noticed in Au@AgNRs structure, which exhibits broad absorption spectrum, sufficiently overlapping with that of dye Rhodamine B (RhB). Au@AgNRs significantly enhance the tunable spectral behavior through localized electromagnetic field and scattering. The random lasing in Au@AgNRs provides an efficient coherent feedback for random lasers.

## Background

The research on plasmonics has led to extensive applications in the field of optoelectronics such as light emitting diodes, waveguides, and nano-lasers, due to the unique property known as localized surface plasmon resonance (LSPR) exhibited by metallic nanostructures [1–4]. In studies based on spontaneous emission, both fluorescence enhancement and quenching, have been observed for fluorophores in the vicinity of metallic nanostructures [5–7]. The fluorescence enhancement and radiative–nonradiative transitions of fluorophores are both found to be strongly dependent on the separation between fluorophores and metallic nanostructures [5]. In random lasers, gain medium is strongly dependent on the scattering strength [8, 9] and light interact with disordered amplifying media in such systems [10, 11]. The scattering is mainly caused by dielectric or metallic scattered light. This mechanism gives to resonating structures with a high quality factor (Q factor). The phenomena of random lasing in some other systems such as nanoparticles, [12] conjugated polymer films, [13] organic dye-doped gel films, [14] suspensions containing laser dyes, silver nanoparticles, [15, 16] and coherent feedback, dielectric materials with high refractive index TiO_2_, and ZnO have also been studied [17–19].

Metal nanoparticles (MNPs) play an important role in spectral narrowing. MNPs have much larger scattering cross section than that of dielectric NPs with the same dimensions. MNPs are enriched with their unique property of surface plasmon resonance (SPR) that may spatially confine light wave near particle surface to give high gain in lasing [20]. SPR position strongly depends on material, shape, size and environment of the NPs. These parameters give spectral tuning of the plasmon resonance to overlap the emission spectrum of the desired active medium. Plasmonic resonances change the local density of optical states to close the NPs and strongly enhance the yield. These NPs can modify the non-radiative and radiative transition rates of nearby dye molecules [21–23]. Generally, lasing dyes have large Stoke shifts between their absorptions and emissions, which could reduce the self-absorption and achieve the lower lasing threshold [24]. The emission intensity may be enhanced in the plasmon assisted random laser by coupling between the dye and localized LSPR of AuNPs [25] provided that there is sufficient overlap between the LSPR spectrum of AuNPs and emission spectra of the dyes. Meng et al. found enhanced emission of coherent random lasing in polymer films embedded with Ag NPs [12]. Ning et al. reported enhancement in the lasing effect of Ag encapsulated with Au NRs [26]. In these reports, the enhanced localized electromagnetic (EM) field was considered to be the dominant mechanism for the occurrence of random lasing, especially for small sizes of Ag NPs. The random lasing could be induced by the effects of both scattering and the enhanced localized EM field of metallic nanostructure.

In this paper, we presented the tunable random lasing properties of AuNRs, AuNPs and Ag encapsulated with Au nanorods (Au@AgNRs). Au@AgNRs showed intensive tunable random lasing behavior, due to their broad spectrum and multiple peaks, covering the emission spectrum of RhB and nearly overlap. Our experiments indicate the phenomena of lasing and variation with different excitation powers. The plasmonic effect was optically excited by the second harmonic of the 1064 nm line of a Nd:YAG laser. The samples were pumped by the second harmonic wavelength at 532 nm, 10 Hz repetition rate, and 6 ns pulse duration. A 532 nm notch filter was used to suppress the detection of the scattered excitation light.

## Methods

### Synthesis of Au@AgNRs, AuNRs and AuNPs

Au@AgNRs were prepared by following the procedure as reported in [27, 28]. The Au–Ag bimetallic nanostructures were prepared using following methods: for AuNRs samples, three aliquots (1 mL) of the AuNRs solution were centrifuged at 800 rpm for 15 min and re-dispersed into CTAC solutions (0.08 M) at the same volume 0.12, 0.24 and 0.48 of AgNO_3_ (0.01 M) were subsequently into the three aliquots of the AuNRs solution, followed by the addition of ascorbic acid (AA, sigma Aldrich) solutions (0.1 M), respectively. The volume of the AA solution was half of that of the AgNO_3_ solution for each aliquot. The resultant solutions were kept in an isothermal oven, present at 65 °C for 3 h. The AuNPs (13 nm) were synthesized by using chemical reduction method, which was carried out as follows: 5 mL of 10 mM chlorauric acid (HAuCl_4_.3H_2_O) solution was heated to boiling 100 mL beaker with 90 mL di-ionized water, stirring at 400 rpm. Then, 5 mL of 3.8 × 10^−2^ M trisodium citrate dihydrate (Na_3_C_6_H_5_O_7_·2H_2_O) was added. The solution color changed within several minutes red wine as continue stirring for 15 min [29]. We used 13 nm AuNPs as a seeds for 40 nm particles. 0.2 mL 13 nm seeds + 5 mL 0.5 mM HAuCl_4_ was stirred at 400 rpm then 30μL then 363 mM NH_3_·OH·HCl were added. Size of the AuNPs 40 nm is confirmed by SEM. The substrate was carefully cleaned by piranha solution to improve the wet ability of the surface [30, 31]. The rhodamine B aqueous solution mixed with AuNRs was stirred over night and the film is prepared by casting drop method. We spread the solution by pipette (300 μL) and dried at room temperature. We made the device using following steps, polyvinylpyrrolidone (PVP, M_w_ ~55,000 sigma Aldrich) were used without further treatment. The PVP was dissolved in de-ionized water (DI water) and was stirred for 4 h. RhB dye molecule and NPs solution were mixed together and placed for few hours for stirring. Then prepared the film for laser characteristic.

### Film preparation

The substrate was carefully cleaned in order to improve the wetability of the surface. The silicon substrate (2 × 2 cm^2^) were washed in a mixture containing concentrated sulfuric acid (95-98%) and hydrogen peroxide (40%) (H_2_SO_4_:H_2_O_2_ = 3:1, volume ratio) for 10 min and were treated in an ultrasonic bath containing ammonium hydroxide solution (40%), hydrogen peroxide and de-ionized water with a volume ratio of NH_4_OH:H_2_O_2_:H_2_O = 1:0.6:0.8 for 5 min. Thereafter, the substrates were washed with copious de-ionized water and dried in nitrogen gas flow before use [30]. Small amounts of the solution (300 μL) were put on the substrate and were carefully spread to fully cover on the substrate. The solution is allowed to slowly dry at room temperature.

## Results and discussion

**Fig. 1:**
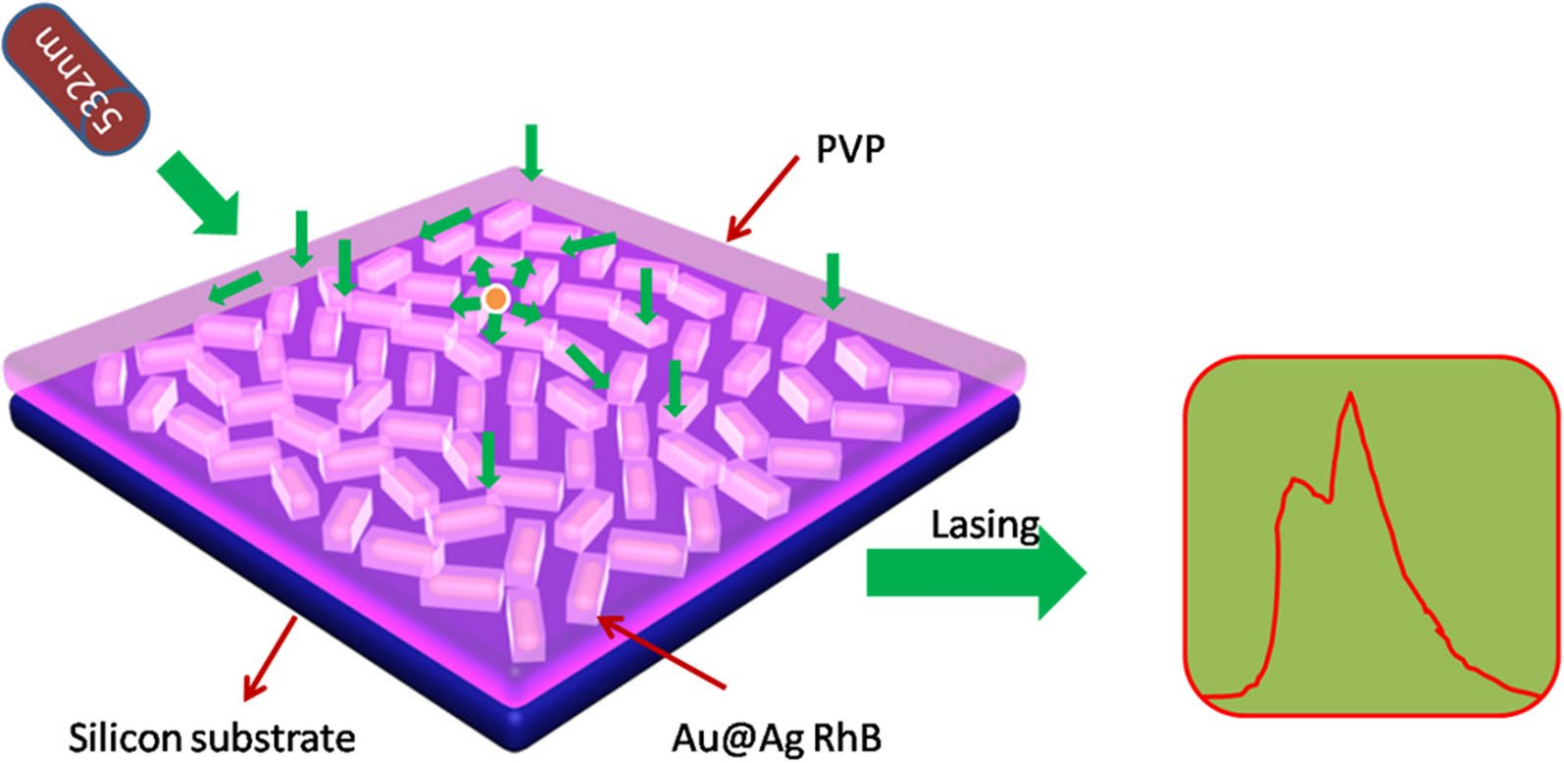
Schematic sketch of the spectral narrowing device and the emission spectrum

A typical sketch of the fabrication of film and experiment are shown in Fig. 1.

**Fig. 2:**
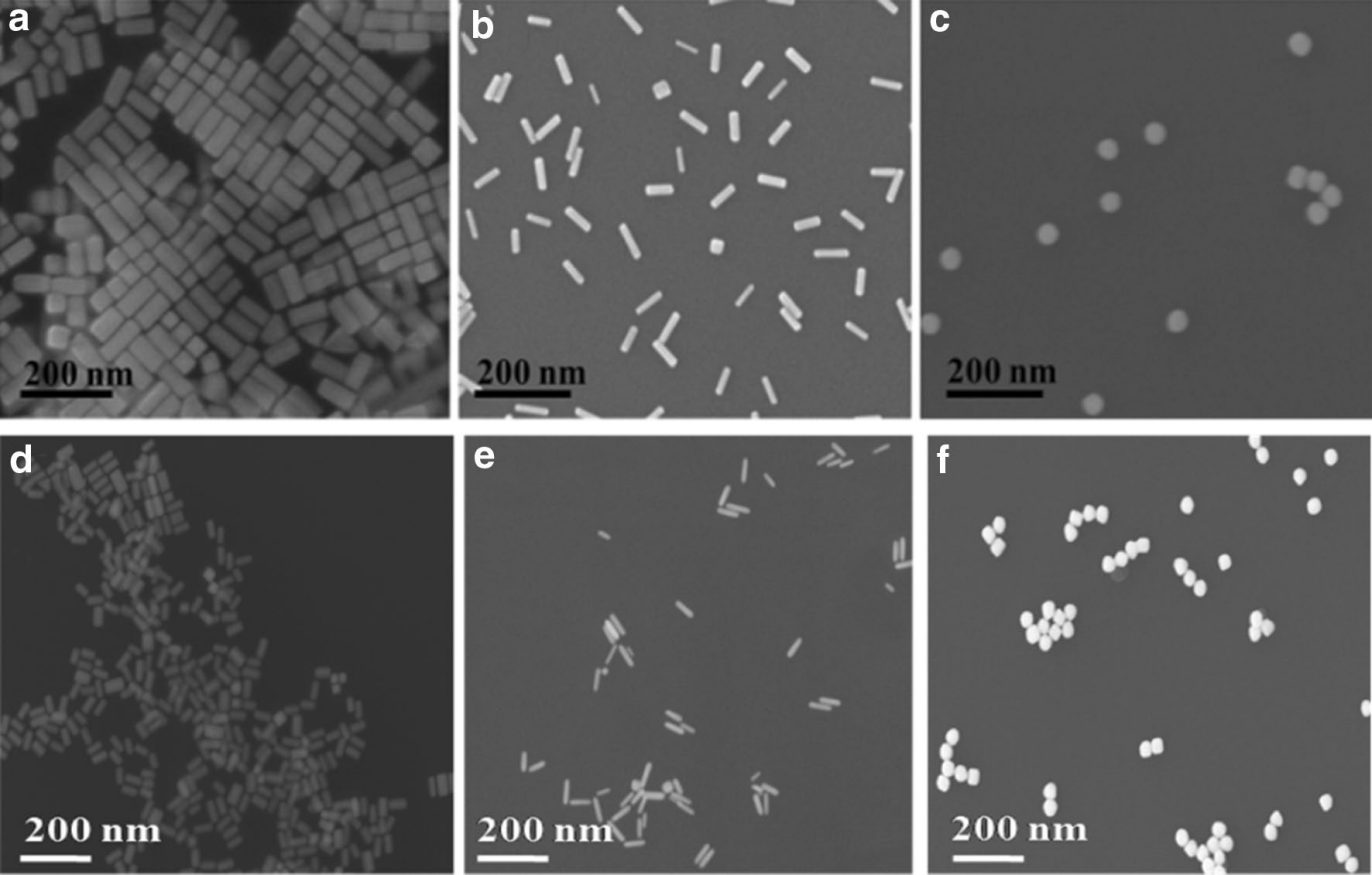
Scanning electron microscopic (SEM) images of Au NPs without dye capping: **a** Nanorods shaped Au@Ag **b** AuNRs **c** AuNPs (*spheres*), with dye **d** Au@AgNRs **e** AuNRs and **f** AuNPs

**Fig. 3:**
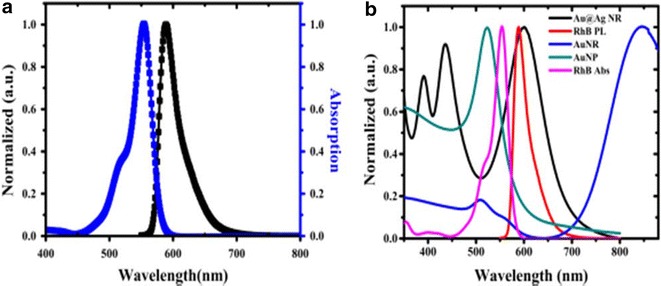
**a** Absorption and photoluminescence (PL) spectra of RhB dye and **b** LSPR spectra of AuNRs, AuNPs and Au@AgNRs with the absorption and emission of RhB

Scanning electron microscope (SEM) of Au@AgNRs, AuNRs & AuNPs has been shown in Fig. 2. All nano structures are quite uniform with size and shape. It is clear from Fig. 2a that the average length of AuNRs is found to be 86 ± 6 nm and thickness of Ag around AuNRs is 42 ± 6 nm. The length of AuNRs is observed to be ~60 nm and width is 12 nm (Fig. 2b). The sizes of AuNPs are found around 40 nm. Figure 2d, e and f are shown SEM after film fabrication. AuNPs are very easy to get the aggregation. To avoid the aggregation we continued stirring then made the film. The absorption and photoluminescence (PL) behavior of RhB are clearly shown in Fig. 3a. Figure 3b shows absorption spectrum of Au@AgNRs. The LSPR peak of Au@Ag NRs has been observed to have four peaks (341, 387, 435, and 597 nm). RhB dye used an acceptor which can be explained by Förster elegant theory (Förster resonance energy transfer, FRET) [32–35]. We have shown comparative study of absorption spectrum AuNRs, Au@AgNRs and AuNPs and emission of RhB dye molecules. Absorption spectra of the Au@AgNRs exhibited the multi-LSPR peaks and broad spectra which have a sufficient overlap with the emission spectra of RhB.

**Fig. 4:**
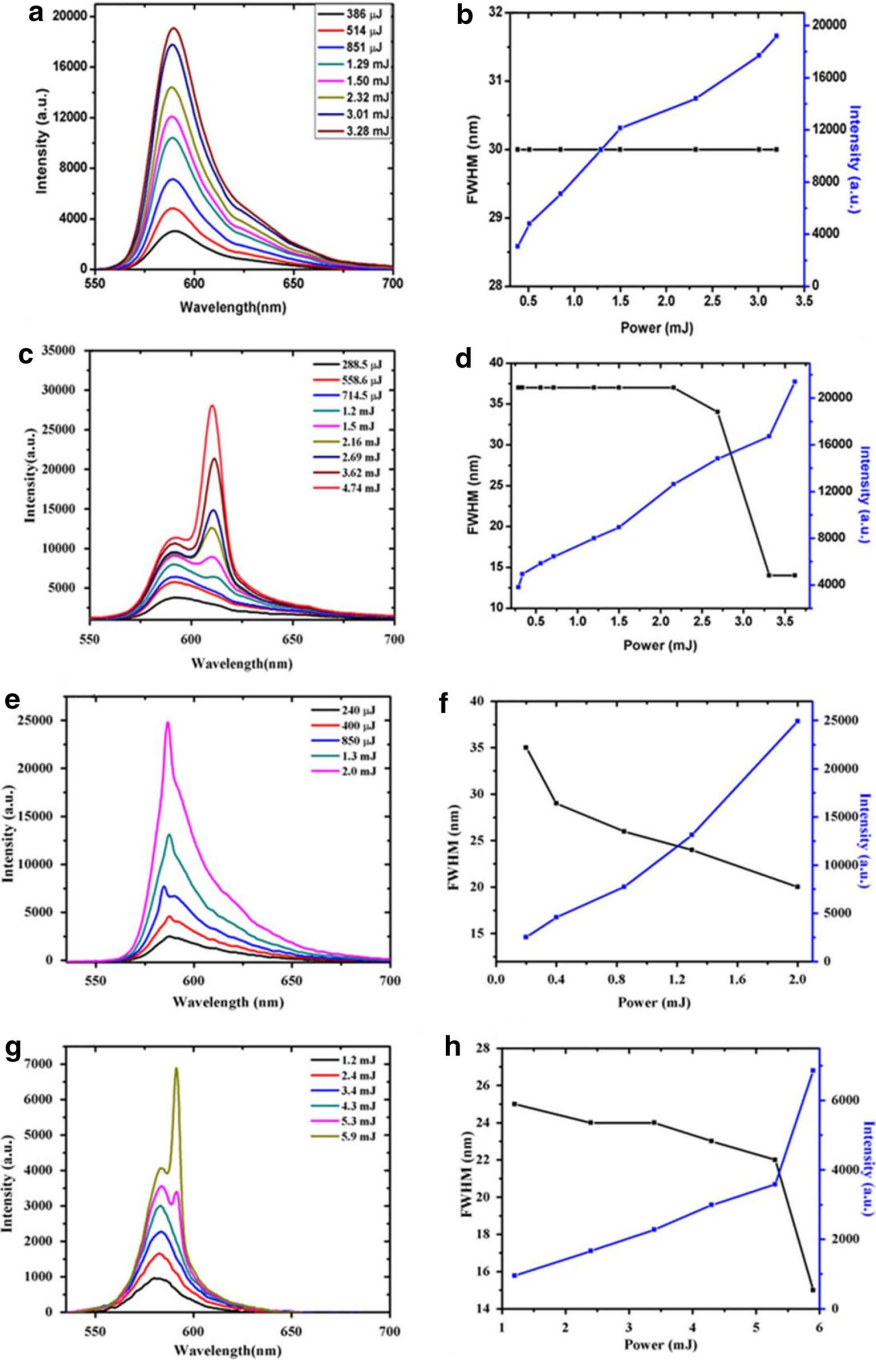
Emission characteristics of thin film contained RhB and gold nanoparticles of different shapes. For each film spectra were recorded with different laser powers, **a** PVP RhB emission spectrum **c** Au@AgNRs showed the spectral narrowing behavior at 1.2 mJ e. AuNRs @ 400 μJ and** g**. AuNPs showing spectral property at high power 5.3 mJ. The corresponding figures **b**, **d**, **f** and **h** are showing the intensity vs. FWHM of the emission spectrum with varying pump powers

Figure 4 shows the luminescence behavior of different samples excited by 532 nm wavelength. PVP RhB film has no spectral behavior at high excitation power. We recorded the spectrum at different powers. We collected the emission spectrum of PVA RhB film as a reference (Fig. 4a). We observed the random lasing in different shapes of gold nanostructures, prepared by casting film deposition method on silicon substrate. Edge-emission spectra of the gain medium with MNPs are shown in Fig. 4c. As described in previous reports [36], the net gain medium film exhibits an obvious lasing behavior. We performed measurements by varying the excitation power from low to high values. MNPs are capped with dye molecules and covered with PVP film are found to excite at high power, which can be explained by FRET [32] and surface energy transfer (SET) [35]. Fluorophores involved in resonance energy transfer affect the spectral properties of the donor and acceptor. MNPs can affect the radiative rate of fluorophores [33, 34]. Modification of the fluorophores’ radiative rate by the metallic structures can lead to enhanced fluorescence intensity [7], and the change in the fluorophores’ radiative rate can be explained in terms of the coupling of the molecular and NPs dipoles. Constructive interference of the dipoles has led to the increased radiative rate and resulted possible enhancement of fluorescence intensity. Radiative and non-radiative decay rates are dependent on the fluorophores’ dipole relative to the particle surface [37]. Dye molecules could be excited simultaneously to higher energy bands and de-excited with an emission. However, the average power absorbed by each dye molecule is very small as compared to spot illumination (with the same pump power). Each molecule will de-excite at a longer wavelength. When the pump power is increased, the radiative transition probability is enhanced at the shorter/longer wavelength end of the spectrum creating a shift in the spectrum towards blue/red wavelengths. Power-dependent spectral peak shifts with different sizes of particles have been reported previously by some other groups [38].

Au@AgNRs exhibited broad spontaneous emission spectra with full width half maximum (FWHM) about 38 nm at low pump power (288.5 μJ). Once the excitation energy becomes large enough, the emission spectrum became much narrow with FWHM 14 nm at 3.62 mJ laser power (Fig. 4c). Intensity vs. FWHM variation of emission spectrum with different laser powers has been shown in Fig. 4d. Red shift observed due to locally enhanced field of gold nano structures, when dye molecules are accumulating on the outer surface of the gold nano structures. [39]. Emission intensity is found to linearly increase with the laser pump powers. On the other hand AuNRs has shown blue shift in our experiment. AuNRs showed the spectral narrowing at 587 nm at 850 μJ in Fig. 4e. Figure 4f, the pump power behavior corresponds to FWHM and intensity. In the case of AuNPs, we got the spectral narrowing @ 591 nm at very high laser power as compared to Au@AgNRs and AuNRs shown in Fig. 4g. We observed random lasing at high pump power. Enhanced localized electromagnetic field (EM) in the vicinity of metal nanostructures may enhance the density of pump light available for the gain media, and consequently may increase the probability of the dye molecules that are to be excited simultaneously to the higher energy levels. AuNPs can affect the radiative rate of a fluorophores [21, 40]. Some research groups reported lasing efficiency enhanced by metallic NPs [41–43]. There are two kinds of mechanism: (1) Enhancement of localized EM field in the vicinity of metal NPs and (2) Enhancement of scattering strength [41]. Increasing the quantum yield of the gain media will depend on the degree of the overlap between the LSPR spectra and the emission of the RhB. When metallic NPs are excited resonantly, they scatter the energy of emitters with the greater scattering cross sections, and then easily lead to the occurrence of spectral narrowing random lasing. We observed that Au@AgNRs absorption have the most sufficient overlap with emission of RhB. Enhancement in the local field is rather moderate for gold nanospheres because the losses dominate over a possible gain due to feedback from multiple scattering events. Nanorods efficiently scatter the photons emitted by the RhB and more over the high local field provide an additional excitation enhancement of the molecules. This can be reason to significant increase in the effective emission rate [44]. In this work we found the best random lasing behavior on Au@AgNRs structure.

**Fig. 5:**
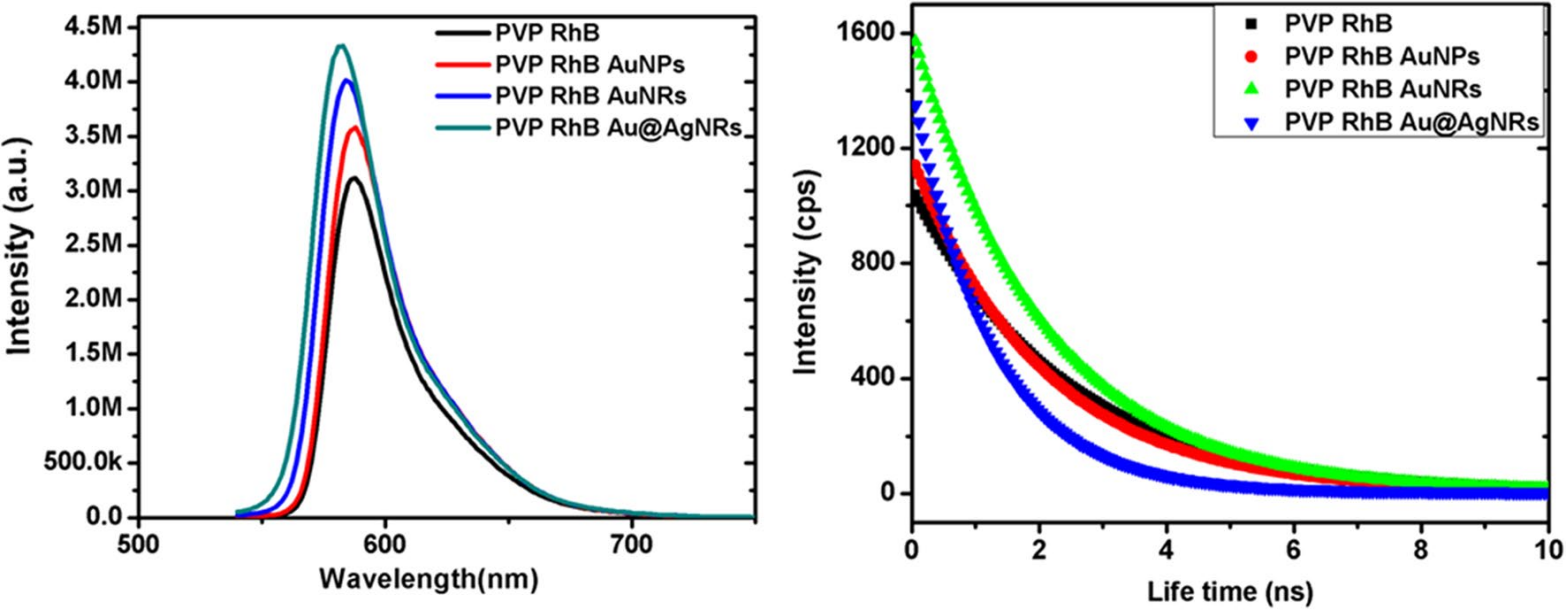
fluorescence spectrum of PVP RhB and different shape of gold nano structures (AuNPs, AuNRs and Au@AgNRs) (*right*) Life time of PVP RhB and mixed with AuNPs, AuNRs and Au@AgNRs

PL measurements performed by fluoromax-4 spectrofluorometer instrument. PVP RhB PL intensity is very less as compare with gold nano-structures clearly seen in Fig. 5.

We used time correlated single photon counting (TCSPC) for this experiment. Time-resolved measurements were performed by Nano LEDs (455 nm; FWHM < 750 ps) with repetition rates between 10 kHz and 1 MHz were used to excite the sample. We were used IBH Data Station Hub photon counting module and data analysis. The PVP RhB lifetime was found 2.28 ns, PVP-RhB-AuNPs 2.10 ns, PVP-RhB-AuNRs 2.08 ns and PVP-RhB-Au@AgNRs 1.59 ns observed in Fig. 5 (right). Fluorescence life time is an intrinsic molecular property.

**Fig. 6:**
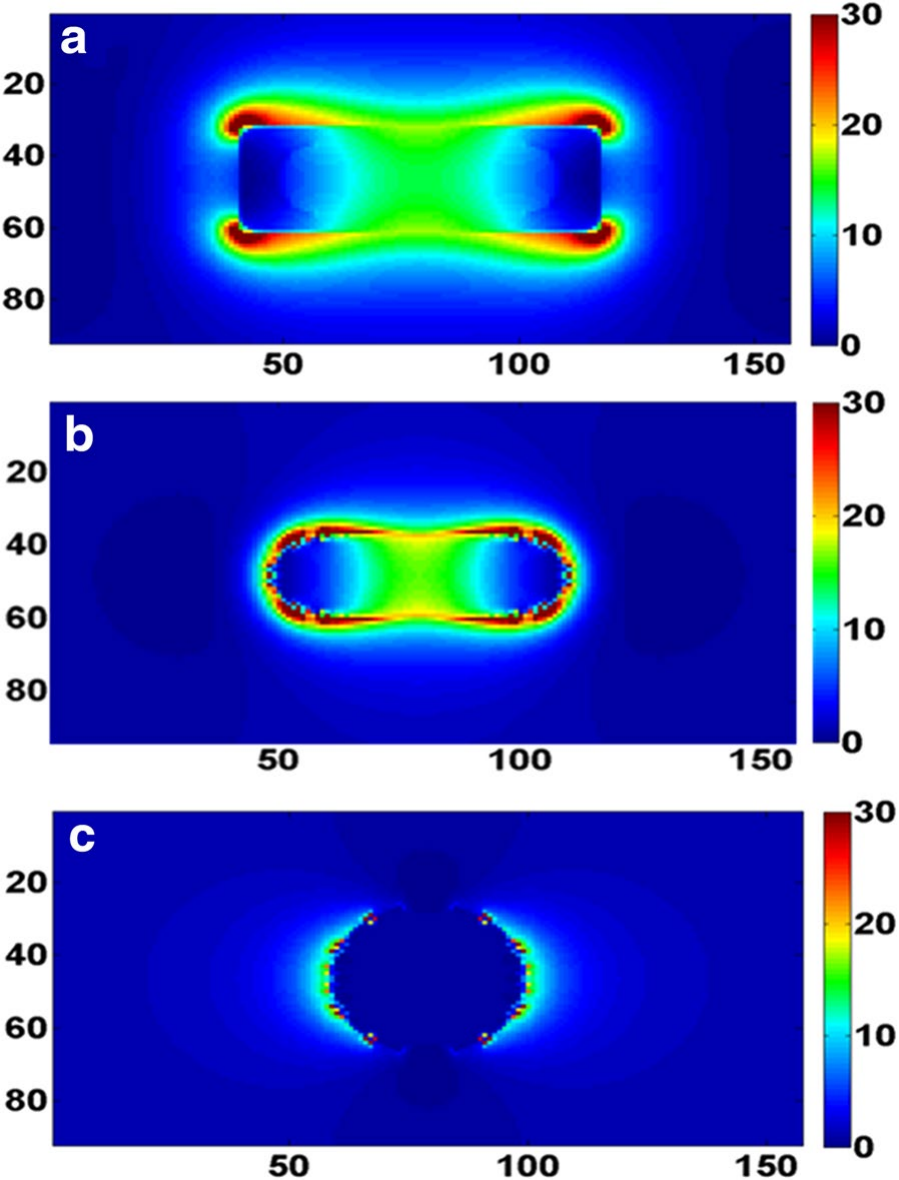
Distribution of electric field normalized to the incident wave at the wavelengths of 611 nm, **a**, **b** and **c** Au@AgNRs, AuNRs, and AuNPs

Further, we confirmed the surface plasmonic effect on metallic nanostructures (Au@AgNRs, AuNRs and AuNPs) (Fig. 6a, b, c). Theoretical optical properties were calculated by the finite difference time-domain (FDTD; Lumerical Solutions, Inc.). The electric profiles of Au@AgNRs strongly affect the local surface electromagnetic field. Au@AgNRs has unique plasmonic characteristic and broad spectra. EM field focalized at the corners or the edges of metal nano structures. Then, we observed very large enhancement factors of the electric field. The FDTD simulation of the electric-field distribution of the Au@AgNRs, AuNRs, and AuNPs with emission light wavelengths at 611 nm is shown in Fig. 6. The color scale indicates the electric field enhancement factors, normalized to the incident wave. It is found that the electrical field of the Au@AgNRs obviously enhanced compared with that of the Au NRs and Au NPs. At the same time, we find that the electrical field of the Au NRs is stronger than that of AuNPs. The simulation confirmed the unique local field enhancement of the Au@Ag NRs, which plays an important role in plasmonic enhanced lasing.

## Conclusion

In this work, we have investigated a tunable random lasing behavior of plasmonic nanostructures (AuNRs, AuNPs and Au@AgNRs) capped with RhB dye. We observed that the plasmonic effect of Au@AgNRs have significantly improved the lasing behavior of the gain medium and showed the best property in comparison of AuNPs and AuNRs. The broader absorption and multiple peaks of LSPR of Au@Ag NRs overlap with both absorption and emission spectrum of the donor–acceptor of the gain medium. These result in the enhanced spectral behavior by the effect of both localized electromagnetic field and scattering. The random lasing in Au@AgNRs provides an efficient coherent feedback for random lasers. This study provides a new approach to achieve the random lasing by tuning the LSPR spectrum of the metallic nanostructures.
